# Supplementary figures and images for: Human newborns form musical predictions based on rhythmic but not melodic structure
Source: PLoS Biol. 2026 Feb 5;24(2):e3003600. doi: 10.1371/journal.pbio.3003600 (PMC12875487; doi:10.1371/journal.pbio.3003600)

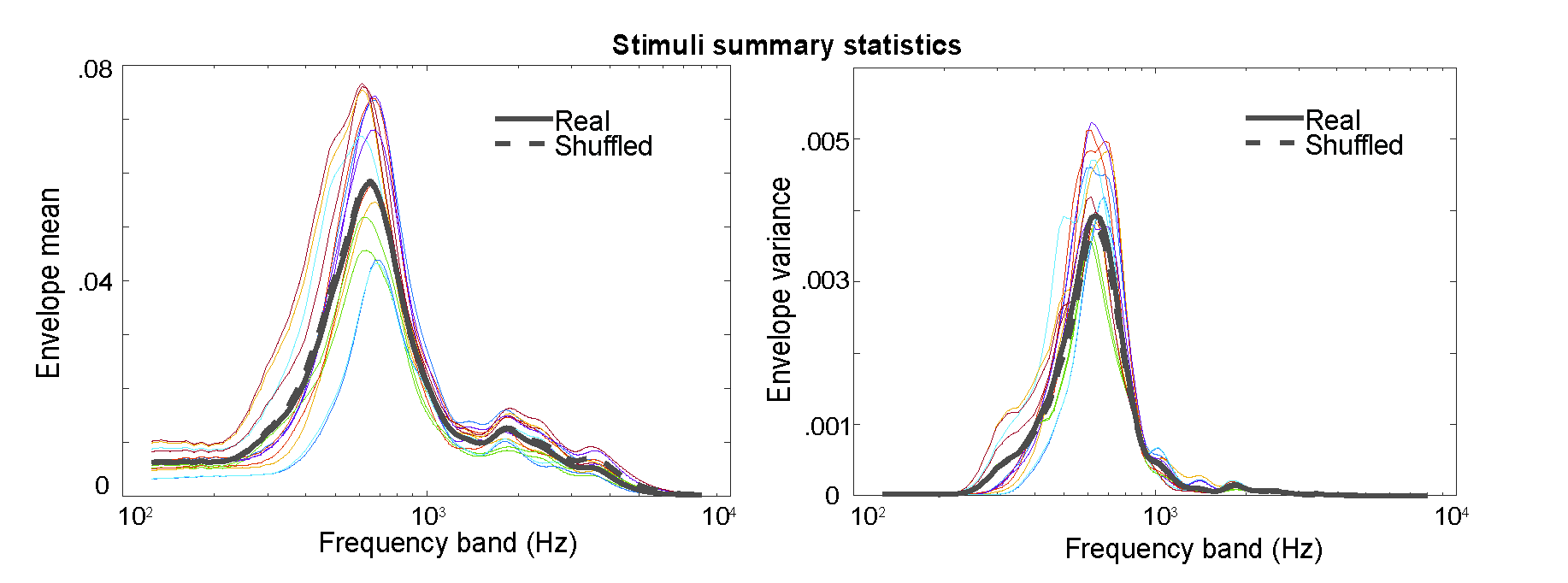

Supplement: S1 Fig — To extract the envelope associated with each frequency band, we bandpass-filtered the musical stimuli into 128 logarithmically spaced frequency bands ranging from 100 to 8,000 Hz using a gammatone filter bank. We then computed the amplitude envelope of each band as the absolute value of the Hilbert-transformed signal over time. The envelope mean (left) and variance (right) are shown as a function of frequency band. Thin lines represent individual melodies (real and shuffled), while thick lines indicate the average across melodies for Real (solid line) and shuffled (dotted line) conditions. Note that the real and shuffled conditions show comparable envelope means and variances. See S5 Data. (TIF) [file pbio.3003600.s001.tif]

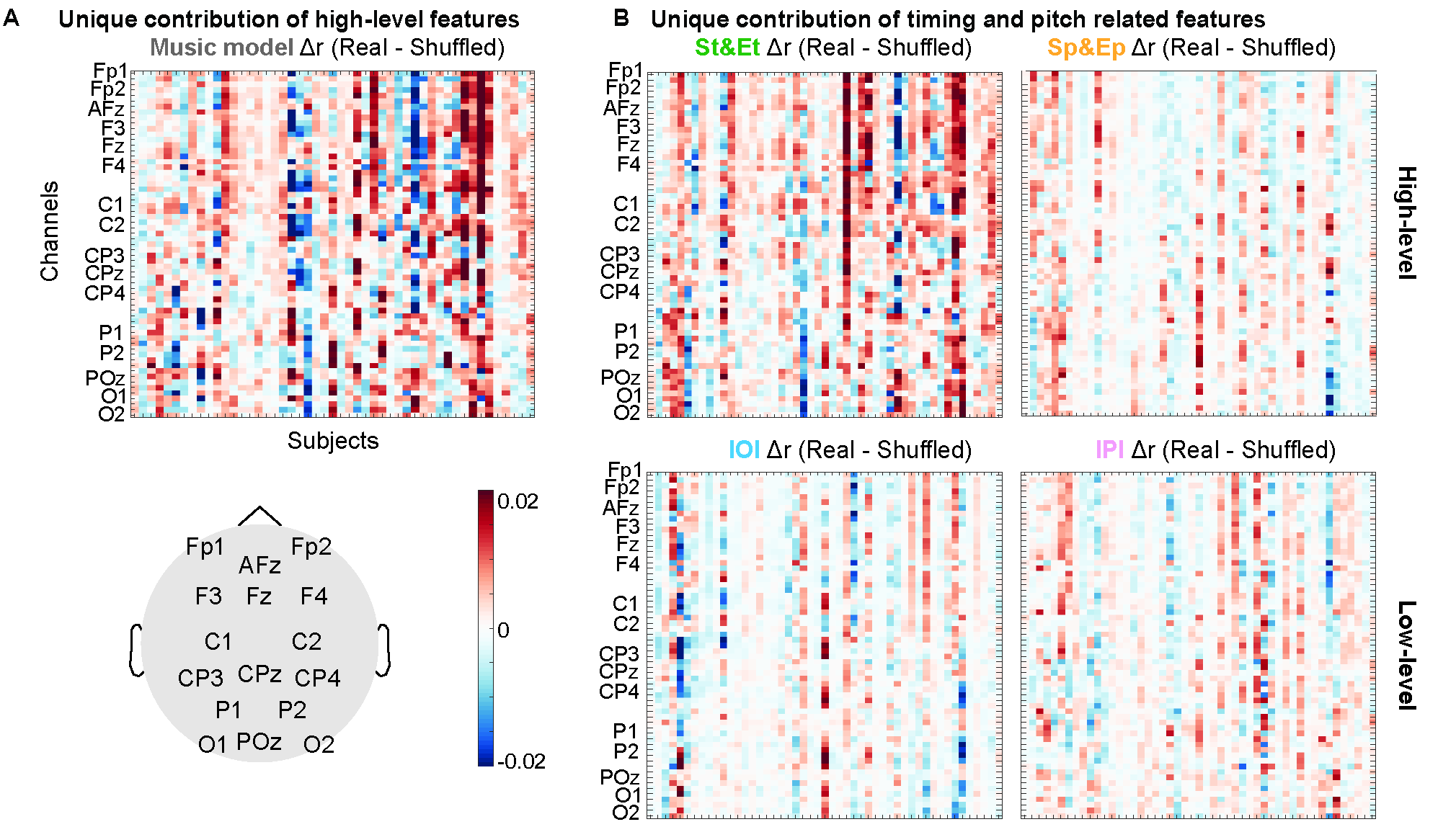

Supplement: S2 Fig — The 2D matrices display electrodes on the Y-axis and participants on the X-axis, with colors coding the difference in Δr values (full—reduced model) between real and shuffled conditions. Note that positive values (red color coded) indicate a greater contribution of the real compared to the shuffled condition. Near-zero values (white color coded) indicate similar contributions across conditions. (A) Unique contribution of high-level musical features. Between condition difference in Δr (full—reduced model assessing the unique contribution of high-level musical features—St, Et, Sp, and Ep). To facilitate visualization, we display the labels of 16 representative electrodes (out of 63) on the y-axis, along with their corresponding position on the EEG cap (bottom). (B) Unique contribution of timing and pitch-related features. Between condition difference in Δr (full and reduced models separately assessing the unique contribution of St and Et, Sp and Ep, IOI, and IPI). See S6 Data. (TIF) [file pbio.3003600.s002.tif]

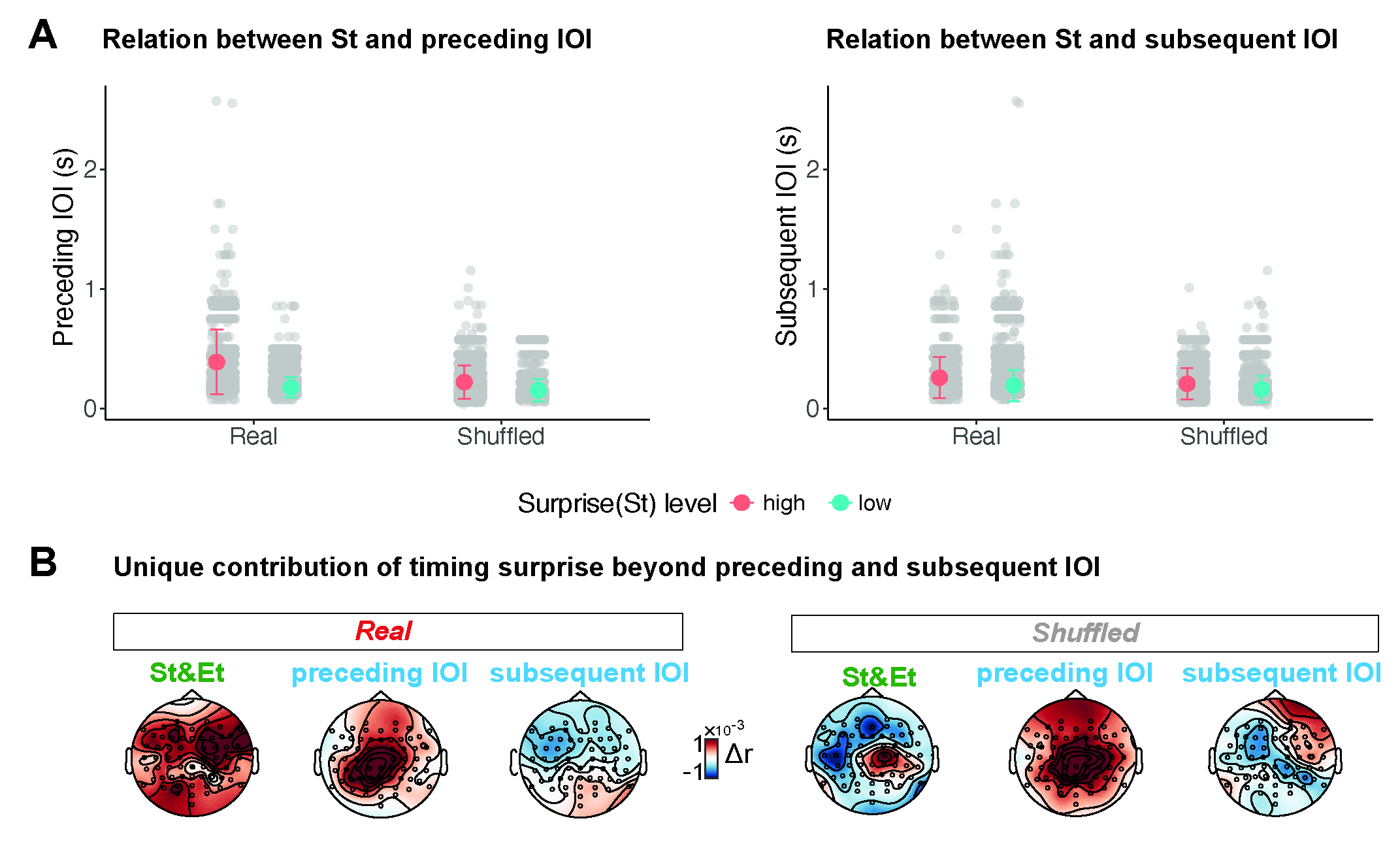

Supplement: S3 Fig — Notes carrying high surprise are often preceded by relatively larger IOIs. A linear mixed model predicting the preceding IOI, with factors condition (real/shuffled) and surprise level (high/low), yielded no main effect of condition (χ2(1) = 1.12, p = 0.29), but a significant main effect of surprise level (χ2(1) = 16.89, p < .001), and an interaction of condition and surprise level (χ2(1) = 18.12, p < .001). This indicates that larger IOIs generally anticipate notes carrying high surprise, more so in real than in shuffled music (left panel). Conversely, the same analysis predicting the subsequent (rather than preceding) IOI, yielded a nearly significant effect of surprise level (χ²(1) = 3.52, p = 0.06) but no main effect of condition (χ²(1) = 0.008, p = 0.90) and no interaction (χ²(1) = 0.70, p = 0.40). This indicates that notes carrying high surprise tend to be followed by larger IOIs, but comparably across real and shuffled music (right panel). See S7 Data. (B) Unique contribution of St and Et is independent of preceding or subsequent IOI. To distinguish neural tracking of rhythm from spurious modulations of event-related potentials (ERPs) attributable to overlapping (i.e., temporally proximal) neural responses, we re-run the main analysis, adding the length of the subsequent IOI as a regressor in the mTRF. We thus run a full model with the following regressors: onset, spectral flux, inter-pitch interval, preceding IOI, subsequent IOI, Sp, Ep, St, and Et. We then computed three reduced models, each randomizing one of the following regressors: 1) St and Et, 2) preceding IOI, and 3) subsequent IOI. The results of this control analysis confirm a unique contribution of St and Et features to the neural response beyond the contribution of subsequent and preceding IOI. The plot shows the topographical maps representing group-average Δr resulting from the difference between the full and the three reduced models across real and shuffled conditions. (TIF) [file pbio.3003600.s003.tif]

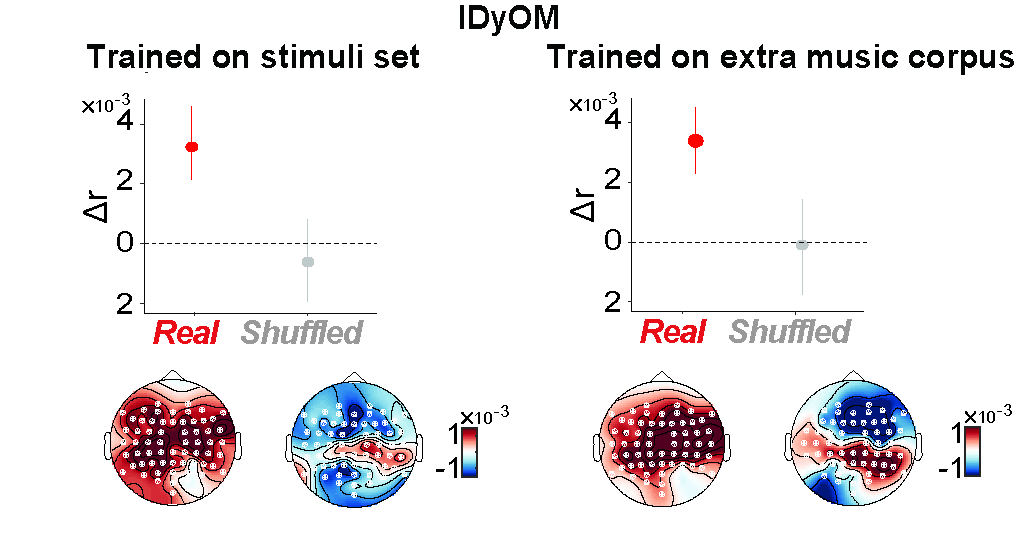

Supplement: S4 Fig — No effects of IDyOM statistical knowledge on EEG prediction accuracy. We compared the effect of deriving surprise estimates by training IDyOM on either the experimental stimuli alone (left panel) or on the experimental stimuli, as well as an additional corpus of Western tonal music (right panel). For each panel, we plot the difference in EEG prediction accuracy (Δr) between the full and the reduced models (randomizing St, Et, Sp, and Ep). Dots represent the grand-average mean Δr computed across all channels and melodies (left top panel, with associated topographical maps) for real (red) and shuffled (gray) music. Error bars represent bootstrapped 95% CI. The absence of differences in predicting neural responses between pre-trained and nonpre-trained model configurations suggests that incorporating pretraining to estimate surprise and entropy values does not enhance the prediction of EEG data. This may be due to the high correlation between the estimates derived from the two IDyOM configurations, leading to similar EEG predictive power. Additionally, it may indicate that Bach’s music contains sufficient rules and statistical regularities, allowing the model to learn these directly from the stimulus set, rendering pretraining on the large music corpus redundant to predict brain signals. See S8 Data. (TIF) [file pbio.3003600.s004.tif]

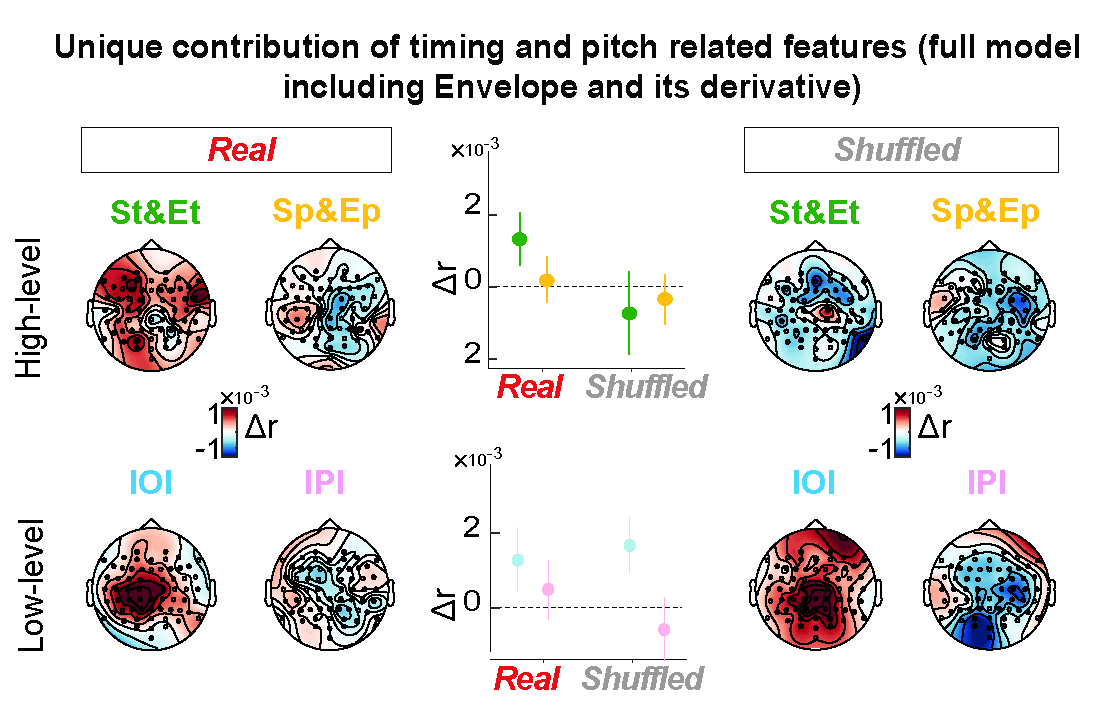

Supplement: S5 Fig — Replication of the results reported in Fig 2C, here adding envelope and its half-wave rectified derivative as predictors in the full model. We repeated the analysis using an enriched acoustic model, thus adding envelope and its half-wave rectified derivative to the already used acoustic regressors (onsets, spectral flux, ITI, and IOI). This additional analysis confirms the robustness of our results: the main findings remain overall unchanged, indicating a unique contribution of the high-level music regressors in the real but not the shuffled music condition, specifically driven by the St and Et regressors. See S9 Data. (TIF) [file pbio.3003600.s005.tif]
