# Supplementary material for: Human newborns form musical predictions based on rhythmic but not melodic structure
Source: PLoS Biol. 2026 Feb 5;24(2):e3003600. doi: 10.1371/journal.pbio.3003600 (PMC12875487; doi:10.1371/journal.pbio.3003600)
Supplement: S1 Table — (DOCX) [file pbio.3003600.s006.docx]

**S1 Table. Characteristics of the experimental stimuli.**

| Melody # | Title | | IOI (s)  (mean, SD, range) | Pitch (MIDI) (mean, SD, range) | Melody Duration (s) | #Notes | Tempo in bpm | Mean envelope (averaged across cochlear channels) | Variance envelope (averaged across cochlear channels) |
| --- | --- | --- | --- | --- | --- | --- | --- | --- | --- |
| 01 | BWV1013 | allemande | 0.15 ± 0.04  (0.75:1.35) | 75.3 ± 5.5  (62:93) | 154 | 1021 | 100 | 0.0172 | 0.6551 |
| 02 | BWV1013 | corrente | 0.17 ± 0.08  (0.75:0.9) | 74.9 ± 5.3  (62:91) | 150 | 890 | 100 | 0.0168 | 0.6882 |
| 03 | BWV1013 | sarabande | 0.38 ± 0.25  (0.21:2.57) | 76.7 ± 5.7  (62:92) | 116 | 300 | 70 | 0.0123 | 0.6370 |
| 04 | BWV1013 | bourrée angloise | 0.25 ± 0.13  (0.19:1.12) | 73.8 ± 4.9  (60:86) | 132 | 528 | 80 | 0.0156 | 0.6637 |
| 05 | BWV 1002 | allemande | 0.38 ± 0.18  (0.07:1.5) | 73.4 ± 6.6  (57:86) | 170 | 619 | 50 | 0.0111 | 0.4545 |
| 06 | BWV1004 | allemande | 0.3 ± 0.13  (0.16:2.55) | 70.6 ± 5.6  (56:86) | 161 | 539 | 47 | 0.0168 | 0.6815 |
| 07 | BWV 1004 | gigue | 0.13 ± 0.04  (0.12:0.5) | 72.0 ± 6.2  (55:86) | 179 | 1351 | 120 | 0.0178 | 0.5821 |
| 08 | BWV 1006 | loure | 0.38 ± 0.19  (0.12:1.5) | 77.6 ± 4.4  (64:85) | 130 | 337 | 120 | 0.0095 | 0.4594 |
| 09 | BWV 1006 | gavotte | 0.27 ± 0.15  (0.11:1.29) | 75.3 ± 4.9  (59:86) | 175 | 641 | 70 | 0.0128 | 0.5889 |
| 10 | BWV 1001 | presto | 0.12 ± 0.03  (0.12:0.72) | 70.0 ± 6.1  (55:86) | 195 | 1603 | 250 | 0.0191 | 0.5766 |
| s_01 | Shuffled | o_01 | 0.15 ± 0.05  (0.04:0.31) | 75.3 ± 5.5  (62:93) | 152 | 1021 | 139 | 0.0165 | 0.5627 |
| s_05 | Shuffled | o_05 | 0.27 ± 0.134  (0.06:0.9) | 73.4 ± 6.6  (57:86) | 166 | 619 | 133 | 0.0126 | 0.4427 |
| s_08 | Shuffled | o_08 | 0.39 ± 0.18  (0.05:1.15) | 77.5 ± 4.4  (64:85) | 133 | 337 | 104 | 0.0098 | 0.4688 |
| s_10 | Shuffled | o_10 | 0.12 ± 0.02  (0.06:0.18) | 70.0 ± 6.1  (55:86) | 193 | 1603 | 163 | 0.0202 | 0.5665 |
