# Supplementary material for: Human newborns form musical predictions based on rhythmic but not melodic structure
Source: PLoS Biol. 2026 Feb 5;24(2):e3003600. doi: 10.1371/journal.pbio.3003600 (PMC12875487; doi:10.1371/journal.pbio.3003600)
Supplement: Attachment — Submitted filename: ResponseToReviewers.docx [file pbio.3003600.s018.docx]

We thank the Editor for granting a three-month extension during the first author’s maternity leave. We are also grateful to the Editors and Reviewers for their thoughtful and encouraging feedback, and for the opportunity to submit a revised version of our manuscript. Below, we provide point-by-point responses (in blue font) to all comments (in black font).

**Reviewer #1:**

This manuscript addresses an important and timely question in developmental neuroscience and music cognition: whether the human ability to anticipate musical structure is already present at birth. The authors use temporal response functions (TRFs) to probe rhythmic and melodic encoding in newborns exposed to real versus temporally shuffled musical stimuli.

The study is built on a particularly rich and valuable dataset — EEG recordings from 49 newborns, an impressive achievement in itself. The main finding — that newborns encode probabilistic rhythmic regularities in real music, but not melodic ones — is highly interesting. It provides compelling evidence that rhythm-based statistical learning is already functional at birth.

We have some concerns regarding the channel selection procedure, which should be reworked.
Moreover, the interpretative sections of the manuscript, especially the conclusion, do not fully do justice to the depth of the results. The discussion often gets sidetracked, particularly by extended considerations of sleep state, while the core implications — the precedence of rhythmic over melodic processing — are underdeveloped. Several rich interpretive avenues (e.g., prenatal experience, cognitive cost of temporal vs. spectral prediction, the nature of the "structured context") are left largely unexplored.

Finally, we are also unconvinced by the relevance of the comparison with non-human primates in this context. While it supports the analytical framework, it does not substantially advance a phylogenetic interpretation, which, in any case, does not seem to be the central focus of the study.

Major Comments

1.      Channel selection procedure: While it is understandable that neonatal EEG signals are inherently noisy, the method used for channel selection—choosing the top 25% based on correlation—appears suboptimal. In particular, it introduces a form of double-dipping in the context of the linear mixed-effects model applied to delta-r (> 0). Indeed, currently the channels are selected based on the highest r-values in the full model, thus the following statistical test comparing full vs. reduced models is not valid. A cluster-based permutation-based approach could offer a more statistically grounded way to assess whether models including high-level features outperform controls. Alternatively, permuting the regressors of interest 1000 times (for instance) would allow identification of channels that respond significantly, rather than relying on an arbitrary percentile threshold.

We thank the reviewer for raising this important point. We would like to clarify that our analysis does not constitute double dipping, and we believe that there may have been a misunderstanding of our procedure. We have now edited the manuscript to clarify (p 14). In particular, our statistical analyses do not involve comparisons between full and reduced models; rather, we compare reduced models amongst themselves. As for the definition of the region of interest (ROI), our approach was to select the top 25% of channels based on correlation values of the full model regardless of condition, hence, this choice is blind to conditions (real vs. shuffled) and to the specific stimulus regressors (Surprise Pitch vs. Surprise Timing) included in the models. This ensures that the ROI definition is fair when comparing conditions and reduced models, as the same set of electrodes is used across all conditions, and therefore the contrasts are not biased by the electrode selection step. We believe that selecting electrodes based on the significance of single regressors, as suggested by the reviewer, would indeed be problematic when statistically comparing across regressors (e.g., Surprise Pitch and Surprise Timing), as it could introduce bias.

Finally, to reassure the reviewer further, using different percentile thresholds for ROI definition did not change the pattern of results. Selecting the top 10% of channels (panel A below) resulted in a main effect of condition (χ²(1) = 10.932, *p* < .001) and an interaction between Condition and TRF model (χ²(1) = 7.503, *p* = .006). Similarly, selecting the top 50% of channels (panel B below) yielded a main effect of condition (χ²(1) = 13.944, *p* < .001) and an interaction between Condition and TRF model (χ²(1) = 10.496, *p* = .001). These robustness checks confirm that our conclusions on predominance of rhythmic over melodic statistical tracking in real but not shuffled music do not depend on the chosen threshold. We have mentioned these confirmatory analyses in the method section (p. 15).

2.      Data visualization in supplementary material: It would also be valuable to include, as supplementary material, a 3D visualization of the delta-r values—participants on the Y-axis, channels on the X-axis, and delta-r represented by color—for both conditions (music and shuffled) and both models (high-level, low-level). This would allow for a direct inspection of the TRF results without averaging over channels or participants, helping assess the robustness of the effects.

We thank the reviewer for this helpful suggestion. Following their recommendation, we have added supplementary visualizations (pasted below) showing the distribution of between condition difference (real – shuffled) delta-r values for each participant and channel and for each reduced model. These plots are provided as 2D matrices representing difference in delta-r across channels (on the Y-axis) and participants (on the X-axis) (this specific axis order was preferable to make the topographical organization of the channels more intuitive and easier to interpret). As expected, these visualizations reveal variability across individuals and electrodes, but they also confirm the same general pattern observed in the averaged results—namely, that the effects are driven by the St&Et model in the real condition, and are most consistent across a set of central electrodes. We have added these plots in supplementary material, Figure S2.

**S2 Fig. Difference in prediction accuracy (Δr) between real and shuffled condition across participants and electrodes for each reduced model.** The 2D matrices display electrodes on the Y-axis and participants on the X-axis, with colours coding the difference in Δr values (Full – reduced model) between Real and Shuffled. Note that positive values (red colour coded) indicate a greater contribution of the real compared to the shuffled condition. Near to zero values (white colour coded) indicate similar contribution across conditions. **A) Unique contribution of high-level musical features**. Between condition difference in Δr (full - reduced model assessing the unique contribution of high-level musical features – St, Et, Sp and Ep). To facilitate visualization, we display the labels of 16 representative electrodes (out of 63) on the y axis, along with their corresponding position on the EEG cap (bottom). **B) Unique contribution of timing and pitch-related features.** Between condition difference in Δr (full and reduced models separately assessing the unique contribution of St and Et, Sp and Ep, IOI, and IPI).

3.      Acoustic model improvements: The low-level acoustic model should be refined. Including at minimum the amplitude envelope and its half-wave rectified derivative would align better with known auditory encoding mechanisms and better capture music-related acoustic dynamics. These features have been shown to strongly modulate auditory responses in both music and speech and may coincide with tempo information in the stimuli. See, e.g., Di Liberto et al., eLife 2020 or Robert et al., bioRxiv 2024 (see below for references).

Our choice of using spectral flux as the core feature of the low-level acoustic model follows directly from prior work in the field, including our own previous studies (Bianco et al 2024 Curr. Biol.) and related work (e.g., Weineck et al., 2021 eLife, Bigand et al 2025 JN). Notably, Weineck at al. (2021) directly demonstrated that spectral flux provides higher prediction accuracy than the amplitude envelope or its derivative of musical stimuli. Indeed, spectral flux is mathematically very close to the half-wave rectified derivative of the envelope, but with the advantage of being more tightly linked to temporal changes in spectral content and thus to rhythmic-related information in music.

Despite this, in line with the reviewer’s suggestion, we have repeated the analysis using this enriched acoustic model, thus including onsets, spectral flux, ITI, IOI, plus Envelope and its half-wave rectified derivative. These additional analyses confirm the robustness of our results: the main findings remain overall unchanged, indicating a unique contribution of the high-level music regressors in the real but not in the shuffled music condition, specifically driven by the St and Et regressors. For reasons of methodological consistency with our earlier work, we would prefer to keep spectral flux as the primary feature of the low-level model in the main text, while reporting the additional control analysis (with envelope + derivative) in the Supplementary Material, Figure S5.

**S5 Fig. Control analysis. Reproduction of Fig 2C, here adding Envelope and its half-wave rectified derivative as predictors in the full model.** We repeated the analysis using an enriched acoustic model, thus adding envelope and its half-wave rectified derivative to the already used acoustic regressors (onsets, spectral flux, ITI, IOI). This additional analysis confirms the robustness of our results: the main findings remain overall unchanged, indicating a unique contribution of the high-level music regressors in the real but note shuffled music condition, specifically driven by the St and Et regressors.

4.      Rewriting the conclusion: The current conclusion includes some redundancy and underplays the main theoretical contributions. Rather than focusing so heavily on the impact of sleep state, the authors should more directly address:

o       Why rhythm may be more predictable than melody (e.g., prenatal exposure to rhythmic patterns through maternal physiology; possible cognitive prerequisites for pitch processing…).

See e.g. Dehaene et al., 2015, Neuron for leads on this topic.

Moreover, the interpretative sections of the manuscript, especially the conclusion, do not fully do justice to the depth of the results. The discussion often gets sidetracked, particularly by extended considerations of sleep state, while the core implications — the precedence of rhythmic over melodic processing — are underdeveloped. Several rich interpretive avenues (e.g., prenatal experience, cognitive cost of temporal vs. spectral prediction, the nature of the "structured context") are left largely unexplored.

We have followed the reviewer’s suggestion, and, in the revised version, we developed the discussion regarding the precedence of rhythm over melody and reduced the section dedicated to considerations on sleep effects. We have also added a paragraph discussing potential neural implementations of rhythmic and melodic expectations, citing the work by Dehaene et al. (2015, *Neuron*) in that context.

p. 8:

*“What could underlie such precocious rhythmic abilities? A potential candidate is the fetal sensory environment, which is characterized by the prominence of biological rhythms. This includes auditory stimulations (e.g., the mother’s heartbeat [4]), as well as vestibular stimulations (e.g., associated with the regular pace of maternal gait [68–70]). An alternative possibility is that newborns developed such predictive skills through exposure to musical input during gestation [71]. This hypothesis, however, appears not to be supported by our supplementary analysis, demonstrating that estimating surprise values using a model pre-trained on a large musical corpus produced similar results (S4 Fig). Thus, fast statistical learning throughout the stimuli set provides a more parsimonious explanation. This is generally consistent with the existence of an inborn automatic statistical learning mechanism for sequence processing [72], and in line with recent EEG evidence of the neonates' ability to rapidly learn transition probabilities across different attributes of speech [73,74]. While we could not manipulate prenatal musical exposure, future research should systematically manipulate it under the hypothesis that greater musical exposure would lead to stronger neural encoding of musical expectations.”*

p. 9:

*“Such generally weak pitch encoding may stem from the fact that fetal hearing is heavily low-pass filtered in the womb [79], resulting in substantial attenuation of pitch details during gestation and slower maturation of pitch sensitivity. This is consistent with immature frequency-specific pathways and coarse frequency tuning at birth [80], as well as immature temporal resolution of different tones (see evidence from 6-month-old infants [81]). This factor, combined with the greater complexity of our stimuli compared with previous work, might explain the limited melodic tracking we observed.”*

p. 9:

*“The dissociation observed between rhythmic and melodic statistical tracking might stem from their independent yet complementary neural implementations – respectively relying on temporal and content-based signaling along the cortical hierarchy [83–85]. This separation grants flexibility, allowing the brain to weigh predictive signals by their reliability to optimize sequence perception [6]. Our findings suggest that the weighting of these two predictive processes is shaped by developmental refinement, with rudimentary pitch encoding at birth, eventually becoming as robust as temporal encoding later on. It is also possible that these two processes are differentially vulnerable to vigilance states. According to this hypothesis, during sleep, timing is favored over pitch because it is more salient and potentially linked to survival-relevant cues [75]."*

o       What exactly distinguishes the "real" from the "shuffled" music, beyond surprise values? Could the authors provide concrete descriptions or statistical measures of this structured context? See, e.g., the work of McDermott.

By shuffling the pitches and IOIs of the real melodies we aimed to manipulate the overall predictability, estimated as surprise values, but maintaining other properties unchanged or at least similar. Therefore, musical melodies composed by Bach contain the regular melodic and rhythmic patterns typically found in tonal western music. In contrast, the shuffled stimuli lack comparable predictability in pitch or timing (including a weak sense of musical beat), despite being acoustically similar. Indeed, also the analysis of summary statistics suggested by the reviewer (as in McDermott et al 2011 Neuron paper), shows that real and shuffled conditions have similar mean envelope and variance (see solid and dotted line in the plots below; coloured lines represent individual melodies).

We now mention this in the revised version (p 3), add the plot in the supplementary material (Fig. S1) and report mean and variance of the envelope averaged across cochlear channels for each melody in S1 Table.

**S1 Fig. Summary statistics (mean and variance of the amplitude envelope across frequency bands) of stimuli.** To extract the envelope associated with each frequency band, we bandpass-filtered the musical stimuli into 128 logarithmically spaced frequency bands ranging from 100 to 8,000 Hz using a gammatone filter bank. We then computed the amplitude envelope of each band as the absolute value of the Hilbert-transformed signal over time. The envelope mean (left) and variance (right) are shown as a function of frequency band. Thin lines represent individual melodies (real and shuffled), while thick lines indicate the average across melodies for Real (solid line) and shuffled (dotted line) conditions. Note that the real and shuffled conditions show comparable envelope means and variances.

o       Whether newborns' predictions are based on prior exposure (e.g., in utero learning) or fast learning during the experiment. If the IDyOM model is trained on the same material as the infants hear, this distinction is essential and warrants more discussion, possibly including references on short context auditory learning in newborns.

We thank the reviewer for rising this important point about rapid learning. We acknowledge this is a limitation of our study as it was not designed to test expectations based on rapid statistical learning vs long-term based. Given that foetuses are somehow exposed to structured temporal patterns in utero, our results could reflect both fast, within-session learning—analogous to an incrementally trained IDyOM—or rely on longer-term exposure—analogous to a pretrained IDyOM. Indeed, pre-training or not pre-training IDyOM on a larger corpus produced similar results, suggesting that pretraining on a large corpus adds little explanatory power for predicting newborns’ brain responses. While this point was previously mentioned in the Methods, given its relevance, we now integrate it in the discussion (p. 8).

*“What could underlie such precocious rhythmic abilities? A potential candidate is the fetal sensory environment, which is characterized by the prominence of biological rhythms. This includes auditory stimulations (e.g., the mother’s heartbeat [4]), as well as vestibular stimulations (e.g., associated with the regular pace of maternal gait [68–70]). An alternative possibility is that newborns developed such predictive skills through exposure to musical input during gestation [71]. This hypothesis, however, appears not to be supported by our supplementary analysis, demonstrating that estimating surprise values using a model pre-trained on a large musical corpus produced similar results (S4 Fig). Thus, fast statistical learning throughout the stimuli set provides a more parsimonious explanation. This is generally consistent with the existence of an inborn automatic statistical learning mechanism for sequence processing [72], and in line with recent EEG evidence of the neonates' ability to rapidly learn transition probabilities across different attributes of speech [73,74]. While we could not manipulate prenatal musical exposure, future research should systematically manipulate it under the hypothesis that greater musical exposure would lead to stronger neural encoding of musical expectations.”*

o       The study would benefit from a clearer contrast with previous work, particularly regarding the shift from detecting isolated deviants to modeling continuous, event-by-event predictions, and from using simple, sparse stimuli to rich, fast musical input — which may challenge newborns' cognitive processing capacity.

We agree that our work is novel in two respects: (1) the absence of strong deviants, and (2) the presence of both melodic and rhythmic information. We have emphasised this in the introduction (p. 3).

*“Therefore, unlike traditional paradigms, our design directly assesses rhythmic and melodic tracking within a full, ecologically valid stimulus, rather than inferring them from detection of salient irregular sounds.”*

Both aspects, though characteristic of ecological music listening, pose a greater challenge for the brain compared with traditional oddball stimuli. Indeed, the absence of melodic tracking in newborns could reflect the increased complexity of our stimuli compared to the stimuli used in previous oddball-like studies, which often entail much simpler sequences that include a smaller alphabet. In contrast, our observation of robust rhythmic statistical tracking even with complex, fast musical input is notable. We now discuss this at p. 9.

*“Indeed, our musical stimuli (across real and shuffled melodies) were characterized by sequences of multiple pitches (N = 38, mean height = 73.04 ± 6.32; range: 55-93, in MIDI notation), IPIs (N = 29, mean interval size = 4.66 ± .4.12; range: 0-28) and IOIs (N = 30, mean interval size = .19 ± .13; range: .03-2.6) presented at varying tempi and different rhythms. While these features better approximate ecological music listening, they indeed also pose a greater computational challenge for the neonate’s brain compared to traditional oddball paradigms, with constant IOI and large infrequent pitch deviants..”*

Minor Comments

1.      In the introduction, the comparison between newborns and monkeys may confuse the notion of "innate" capacities, particularly as fetuses are already exposed to music and speech. A brief clarification on what "innate" is taken to mean in this context would help.

To avoid confusion, we have removed the word “innate” from the manuscript. Where previously we referred to “innate predispositions”, we now describe these as biological predispositions to learn certain musical features, which captures our intended meaning without implying exposure-independent capacities.

2.      Relatedly, drawing on developmental literature about early temporal processing might provide a more relevant theoretical framework than uniquely the primate comparison.

In the revised manuscript, we have rebalanced the introduction (p. 2) to draw more extensively on developmental studies on temporal processing. Specifically, we now cite key studies showing that temporal processing is a foundational dimension of early auditory cognition, in that the neonates’ brain already shows considerable specialization to different temporal cues in the speech (Cabrera & Gervain, 2020, Sci Adv) and non-speech signals (Telkemeyer et al., 2009, J. Neurosci.)). We also cite complementary evidence from preterm infants indicating that structured temporal input, such as music, can strengthen auditory networks and scaffold later language development (Lordier et al., 2019, PNAS).

3.      Could the authors provide more interpretation of the correlations between stimulus features? It would help understand to what extent they are separable in the model.

In naturalistic materials, such as music, acoustic and statistical dimensions co-vary to a certain extent. For example, large temporal or pitch deviations tend to be unexpected, particularly in structured compositions like Bach’s. This interdependence can introduce ambiguity about which feature truly drives neural responses. However, as shown in Figure 1C, these correlations are small (< 0.3) to moderate (< 0.5), indicating partial but not complete overlap among predictors. Importantly, the variance partitioning approach used here accounts for shared variance across regressors, allowing estimation of their unique neural contributions. Yet, we acknowledge that a more direct way to assess the unique contribution of individual features is through causal manipulation, as demonstrated by the “model-matched” stimulus approach (Norman-Haignere & McDermott, 2018, *PLOS Biology*). We refer to this issue in the methods (p. 13).

4.      Consider comparing model performance (correlation values) with prior EEG TRF studies (e.g., Di Liberto et al., 2020, eLife; Robert et al., 2024, bioRxiv) to contextualize what constitutes a "reasonable" model accuracy.

In our data, the full (AM) model yielded cross-validated correlation values ranging from 0 to 0.08. This is overall lower (due to noisier data in newborns than adults) but comparable to previous EEG TRF studies using similar approaches, such as Di Liberto et al. (2020, *eLife*; range 0–0.15). Using different stimulus modelling might improve the prediction accuracy (mean ≈ 0.2) such as in Robert et al. (2024, *bioRxiv*), but we chose to keep using IDyOM for continuity with our previous work.

We note that absolute correlations and Δr values depend strongly on recording noise and modelling parameters, making direct comparisons across experiments only approximate. We now briefly mention this correspondence in the Results section (p. 5) when first reporting the model coefficients to contextualize the observed effect sizes.

*“Fig 2B (left panel) shows that the full model - including all features - predicted the EEG data with reasonable accuracy across virtually all participants, yielding correlation values comparable to those reported in previous TRF studies (Di Liberto et al., 2020; Robert et al., 2024).”*

5.      The term "reduced model" is potentially misleading, as its size is not reduced but its meaningful content is randomized. Consider rewording to avoid confusion.

We acknowledge the reviewer’s concern but prefer to retain the term *“reduced model”*, as it accurately reflects our analytical approach. We use it in the same sense as Musall et al. (2019, *Nature Neuroscience*), who defined *reduced models* as those in which a given predictor is temporally shuffled to estimate its unique contribution to the full model. Specifically, the reduction refers to the removal of meaningful temporal structure in one variable, not to a decrease in model size. We have clarified this usage in the Methods section (p. 13).

6.      Figure 1D lacks the representation of the full model, making it unclear for readers.

Thanks for the comment. We have edited the graph in Figure 1D to make it clearer.

7.      Typo in Figure 2B (x10^-3 missing on the right axis).

Actually, this is correct. The axes look bigger because of one subject having high gain.

See alternative visualization here:

8.      Typo in Figure 2A: label should read "IPI" instead of "ITI".

Thank you for catching this! The label has been edited.

9.      Regarding ERPs: are unexpected pitch and unexpected timing correlated? A short clarification would be helpful.

The pitch- and time-related surprise values (Sp and St) are only weakly correlated (rho = .17, p < .001), indicating that unexpected pitch and unexpected timing events are processed independently. It is therefore likely that the corresponding ERP responses reflect mostly distinct neural processes. We have added this point to the manuscript (p. 7).

10.     Does musical tempo affect time prediction accuracy? If so, could the authors explore whether infants show a preferred tempo for prediction?

Thanks for the interesting question. The melody-specific prediction accuracy values have now been ranked by tempo (see Figure 2B: Audio8 = 40 bpm, Audio6 = 47 bpm, Audio5 = 50 bpm, Audio3 = 70 bpm, Audio9 = 70 bpm, Audio4 = 80 bpm, Audio1 = 100 bpm, Audio2 = 100 bpm, Audio7 = 120 bpm; Audio10 = 250 bpm). We have also included information about tempi in Table S1, where we provide acoustic characterizations of each melody. Because we do not see a clear association between a specific tempo and prediction accuracy, we have decided not to discuss this issue directly in the manuscript.

11.     Procedure: Why are some melodies heard twice? Could this repetition facilitate learning and influence the results?

Some melodies were repeated to increase the signal-to-noise ratio (SNR) of the EEG signal. This approach is consistent with Diliberto et al. 2020 (eLife), where melodies were repeated three times and no effect of repetition was observed. In the present study, it would be difficult to assess a potential effect of repetition, as the repetitions were not consistent across infants. None of the babies heard the full set of 14 melodies twice (range 14-25), and on average, they had 1.18 (SD = 1.4) repetitions. We added these details in the methods (p. 12).

**Reviewer #2:**

This paper presents results from a novel EEG study with sleeping newborn humans. The babies were presented with normal and shuffled experts of monophonic piano versions of Bach melodies. The authors consider both low level features, like inter-pitch-intervals and inter-onset-intervals as well as "higher level" quantifications of entropy and surprisal (generated from IDyOM) when testing models for predicting infant neural responses using mTRFs. Results suggest that EEG data were accuracy predicted using models that included both low and high level features. High level features added significantly to prediction accuracy but only for real and not shuffled melodies. This effect seems especially driven by high-level timing features but perhaps not high-level pitch features. ERP analyses tell a similar story - notes that are higher in temporal surprise generate a larger P1/P2-ish response than notes low in temporal surprise for real but not shuffled sequences (no such effects with pitch surprisal). The authors compare these findings to re-analyzed data from awake adults (hearing the "real" stimuli) and awake rhesus monkeys (hearing the same "real" and "shuffled" stimuli).

Overall, this is a very interesting and worthwhile study that answers some questions about early rhythm perception, raises some new interesting questions about how predictions are made about pitch vs timing in auditory stimuli, and uses compelling and cutting edge analysis techniques to explore these questions. I particularly like the use of real vs shuffled music. The authors present an interesting and thoughtful discussion.

1. In that discussion, they highlight something I was thinking about as I read the paper - that their comparison to adult and monkey data should be taken with a grain of salt given that the infants were the only group out of the three that were asleep. I agree that this is really important to highlight and appreciate the discussion provided. Is the "unimportance" of pitch here the result of development/experience or related to attentive/inattentive processing? I have to say, if the authors had the means to collect a sample of sleeping adults hearing both the shuffled and real stimuli, that would greatly strengthen the impact of this paper. I also realize that this was already a ton of work and is enough "as is", so if the authors choose to suggest running sleeping adults through this paradigm as a future direction (which they do) they may need to tone down some of their conclusions -  for example, the entire final paragraph of the discussion.

We very much appreciate the reviewer’s comment and could not agree more on this point. Unfortunately, this work was supported by a Fellowship awarded to the first author; the funding is now over, and she has since moved to a new institution. We are currently planning to run such a study through a collaboration with a lab specialized in sleep research.

In the revised manuscript, we explicitly mention that the absence of evidence for melodic expectations in infants may reflect their sleep state. Accordingly, we cannot draw strong conclusions about the phylogeny of melody. Still, we can say that, compared to rhythm, melody may be more susceptible to both postnatal experience (exposure to music/language) and top-down processing. Future studies should explore melodic processing in sleeping adults to clarify whether the absence of melodic expectations is to be attributed to brain immaturity or to the reduction of top-down processing.

According to these considerations, we have toned down the conclusion in the final paragraph (p. 10):

*“Overall, this study provides neurophysiological evidence that tracking rhythmic statistical regularities is a capacity present at birth, whilst melodic tracking might not be, at least with respect to naturalistic musical stimuli such as the ones we used here. Future investigations should assess whether the observed dominance of rhythm over melody reflects state-dependent factors such as sleep or instead marks an early developmental bias that gradually shifts with experience toward the balanced sensitivity observed in adulthood.”*

2. In the introduction, it would be helpful to spend a bit more time explaining probabilistic regularities of melodies. I'm having a hard time understanding how we could possibly make these predictions about pitch without exposure to a musical system. Is the point that we see lower surprisal for pitch if the same kinds of intervals happen again and again within a sequence? This must take at least a bit of time to establish. It would be helpful to clarify this for those who may be unfamiliar with IDyOM/music theory, and it would be very helpful to link readers to your specific set of stimuli (that link to the Bach website was quite a rabbit hole).

Indeed, pitch regularities can emerge even within a single sequence: if certain pitches or intervals occur more frequently than others, listeners can form expectations “on the fly”, without prior exposure to a musical system. This allows some degree of prediction based on statistical regularities within the sequence. We have added a sentence introducing this potential effect in the introduction (p 2).

*“In music, tracking of statistical patterns is largely implicit [7], allowing the brain to anticipate events or patterns that occur more frequently than others based on both recent and past contexts. Expectations, therefore, build on statistical regularities acquired in real time as the current sequence unfolds, and/or retrieved from prior exposure.”*

The stimuli are available for reviewers at the link <https://dataverse.iit.it/privateurl.xhtml?token=670a5a82-3b50-46cf-954b-caa5f8e3bf62>, that was provided under “data availability statement”. To view the repository structure, one must click on 'Tree’ and access the stimuli in the STIMULI folder. Since this is not obvious, we have now specified this in the data availability statement. Upon publication, the repository with data and stimuli will be accessible at doi:10.48557/GMOFYE.

3. There was some discussion in the paper about how the IDyOM model using only this small set of stimuli came up with similar numbers compared to a model using a large corpus. But even still, the first time infants hear a few of these melodies, they can't be expected to already have accrued enough information about pitch regularities to make useful predictions.

Melodic pitch information is mostly filtered out in the womb, especially more so than timing information (something that could be discussed), so even papers showing musical memory from womb to birth likely rely on timing information. Anyway, all that to say, did you consider exploring whether infants begin to rely more on higher-level pitch patterns in the last few trials than in the first few trials?

This is a very interesting and plausible hypothesis. To address it, we conducted an exploratory analysis comparing neural responses to the melodies presented earlier versus those presented later in the experiment. We run the same reduced model comparison as reported in the main analysis by taking the data from the first and last quartile of heard melodies per each subject (real music condition). The results (see plots below) did not reveal significant pitch-related tracking, neither in the early nor in the later trials. It also did not show changes in rhythmic tracking, possibly because our experiment was not designed to test within-session learning effects.

We have also revised the discussion on melodic sensitivity along the lines proposed by the reviewer (p. 9):

*“Such generally weak pitch encoding may stem from the fact that fetal hearing is heavily low-pass filtered in the womb [79], resulting in substantial attenuation of pitch details during gestation and slower maturation of pitch sensitivity. This is consistent with immature frequency-specific pathways and coarse frequency tuning at birth [80], as well as immature temporal resolution of different tones (see evidence from 6-month-old infants [81]). This factor, combined with the greater complexity of our stimuli compared with previous work, might explain the limited melodic tracking we observed. Indeed, our musical stimuli (across real and shuffled melodies) were characterized by sequences of multiple pitches (N = 38, mean height = 73.04 ± 6.32; range: 55-93, in MIDI notation), IPIs (N = 29, mean interval size = 4.66 ± .4.12; range: 0-28) and IOIs (N = 30, mean interval size = .19 ± .13; range: .03-2.6) presented at varying tempi and different rhythms. While these features better approximate ecological music listening, they indeed also pose a greater computational challenge for the neonate’s brain compared to traditional oddball paradigms, with constant IOI and large infrequent pitch deviants. In sum, newborns’ sensitivity to pitch appears far from sufficient for appreciating musical pitch regularities, which likely emerge through maturation and enculturation. According to this view, reports of musical memory from the womb to birth [82] are likely to rely primarily on timing than pitch information, a hypothesis that deserves further testing.”*

4. Real and shuffled stimuli differ broadly in entropy and surprisal. Is there any argument to be made for exploring how entropy and surprisal (regardless of shuffled/real categorization or within shuffled/real categories) relates to mTRF prediction accuracy? What is the value of considering surprisal and entropy separately in this paper? Do they tell us different stories? If not, why not pick one? If yes, the difference and value of presenting both is unclear.

We consider both entropy and surprisal in line with previous work using the same stimuli (Diliberto et al., 2020; Bianco et al., 2024). Importantly, these measures capture complementary aspects of musical structure: entropy quantifies the uncertainty associated with an event itself, whereas surprisal (information content) reflects how unexpected an event is given prior context. Since our focus is on rhythmic versus melodic expectations, including both surprise and entropy predictors allows us to fully represent pitch and timing features in the models, ensuring that we capture neural activity related to both aspects of musical anticipation. We have clarified our rationale in the Results section (p. 3).

*“Surprise and entropy provide complementary characterizations of predictive processing: entropy captures the inherent uncertainty of an event, whereas surprise reflects the unexpectedness of that event given prior context. Including both predictors allows us to fully represent pitch and timing tracking, ensuring that we capture neural activity related to both aspects of musical anticipation.”*

5. These results raise interesting questions about individual differences - thank you for plotting individual data on the figures. Is it worthwhile exploring whether the babies who rely on higher level features the most for timing do the same for pitch? You mention that gestational age may explain some variability here. Do you have that information? Can you test that prediction? It might also be worth citing François et al., 2017, who report that newborn infant EEG responses to continuous song predict language outcomes in toddlerhood.

We indeed explored whether infants who rely more on higher-level features for timing do the same for pitch but found no correlation between Δr of Sp and St (Real condition: Spearman ρ = 0.234, p = 0.105; Shuffled condition: Spearman ρ = 0.209, p = 0.15). This is now reported on p. 6.

*“Second, to rule out the possibility that some, even if not all, infants were able to generate pitch-based probabilistic expectations, we explored whether those infants generating relatively stronger predictions for timing were also generating stronger predictions for pitch. To do so, we correlated Δr across Sp and St but found no significant correlation for either real (Spearman ρ = 0.234, p = 0.105) or shuffled music (Spearman ρ = 0.209, p = 0.15).”*

Similarly, we explored if gestational age predicts better music encoding, but there was no correlation between gestational age and prediction accuracy of the full model (Spearman ρ = 0.183, p = 0.207). This result is reported in the manuscript (p. 5).

*“The intersubject variability was substantial but not explained by gestational age [58] (Spearman ρ = 0.183, p = 0.207), perhaps reflecting the limited variability of this measure in our sample (gestational age mean 279.8 GG ± 6.8, range 257-290 GG).”*

We also added a citation to François et al. (2017) in the introduction (p. 2), when introducing the notion that exposure to structured inputs such as music might interact with later language development.

Minor comments:

-       Can you clarify with the ERP analyses if the results are on Fz, as shown in the figure? Why was this electrode selected? It is being compared to Fcz in adults and monkeys.

Is there an argument for presenting the same electrode in these figures across groups?

We chose Fz as the electrode of interest because FCz is not part of the EEG cap configuration for newborns (see the template here). Furthermore, based on the topographies, the observed effect in infants is more frontal than central (Fig. 2c), justifying the focus on Fz.

-       It might be nice to add more discussion about primate rhythm perception/production in the intro. I was more familiar with behavioural work showing that primates are not good at predictively tapping but learned a bit from this paper about newer work showing that beat perception may still be happening in some primates. Elaborating on how beat perception/productions in humans compared to non-human primates in the intro would be helpful given the phylogenetic arguments in the discussion.

We agree with the reviewer that the discussion of beat perception and production in non-human primates is fascinating, particularly because it remains a debated topic. That said, our focus is on statistical learning based on rhythmic patterns rather than beat perception. In addition, both the editors and two reviewers suggested toning down comparisons between humans and non-human primates, so we feel we should not expand this section further.

-       This paper appears to argue that the predictive power of the IDyOM outputs suggests that infants are attuned to statistical regularities in musical beats. These statements about statistical learning have me thinking about other work by some of the authors here, specifically Háden's 2024 paper in Cognition suggesting that newborn EEG responses to beats do NOT reflect statistical learning. How do these two pieces of work complement each other (or not)?

To avoid misunderstanding, we would like to emphasize two points. First, our current study does not focus on beat perception (we did not directly manipulate nor measure this aspect of timing). Second, the absence of a statistical learning effect on the neural response in Háden et al. (2024) does not imply that statistical learning is absent, but rather that detecting it under those specific conditions was challenging. More importantly, in relation to our findings, Háden et al. (2024) focused on statistical learning of transition probabilities between spectral features of events—what is traditionally referred to as statistical learning. The term “statistical learning” in that paper is therefore not identical to how it is used in the context of IDyOM, where it refers more broadly to learning transition probabilities of any defined musical attribute. In our case, the attribute is the IOI ratio of events, reflecting sensitivity to temporal statistics based on rhythmic patterns. As a result, there is no fundamental contradiction between the two studies—only a difference in how “statistical learning” is defined.

-       Last paragraph of discussion: Social interactions do not begin at 6 months

We agree with the reviewers that referring to 6 months as the “onset” of social interaction and language development is misleading. During the revision, we substantially restructured the discussion and decided to remove that paragraph altogether, as it felt somewhat off-topic and tangential to the main focus of the paper.

-       The links to language presented in the end of the discussion are unclear. I'd argue that speech processing usually relies on temporal cues more than pitch cues unless we are thinking about prosody. But for understanding word meaning and basic communication, why would pitch be prioritized?

We thank the reviewer for rising this point. We agree that temporal cues are fundamental for speech comprehension. However, what we meant was that prosodic pitch cues become essential during early infancy to supporting syntactic parsing, word boundary detection, and the interpretation of communicative intent (van der Burght, 2021; Saffran et al., 1996). In any case, as mentioned in the previous comment, we have removed that paragraph.

**Reviewer #3** (Anne Kösem):

This study utilizes EEG recordings in newborns to examine whether infants can track pitch and rhythmic expectations in musical excerpts. The authors found that encoding of probabilistic rhythmic expectations occurred only in response to real music, as opposed to shuffled music. However, no evidence was observed regarding the tracking of melodic information.

I would like to express my appreciation for the clarity and quality of the manuscript. The study presents a highly relevant topic and is executed with great competence. Furthermore, the methodologies employed are robust and well-founded.

We greatly appreciate the reviewer’s thoughtful feedback and encouragement.

1- My main inquiry pertains to the interpretation of the results related to rhythmic expectations. I am curious if these findings genuinely support the existence of predictive timing mechanisms as the authors suggest. Alternatively, these results might indicate non-predictive mechanisms, potentially arising from the superposition of evoked responses that arrive at different timings. The authors report a significant effect of inter-onset interval on the mTRF, which, in my view, could signify the impact of the evoked response to the preceding sound—possibly occurring as recently as 100 ms prior. Moreover, it is conceivable that the inter-onset interval of the subsequent tone may also affect the mTRF. If, in real music, the inter-onset interval of the next tone is biased in duration under conditions of high surprise versus low surprise, this could further influence the observed results.

We thank the reviewer for raising this important point, and we agree that rhythmic tracking must be distinguished from spurious modulations of event-related potentials (ERPs) due to overlapping responses.

We now report in Figure S3 (also pasted below) that indeed larger IOIs anticipate relatively more surprising notes. A linear mixed model predicting the preceding IOI, with factors condition (real / shuffled) and surprise level (high / low), yielded no main effect of condition (χ2(1) = 1.12, p = 0.29), but significant main effect of surprise level (χ2(1) = 16.89, p < .001), and an interaction of condition and surprise level (χ2(1) = 18.12, p < .001), suggesting that this bias is stronger in real than in shuffled music (Figure S3A, left panel). However, we do not believe that this constitutes an issue for our interpretation of the results because our TRF analyses included the preceding IOI as a regressor, allowing us to separate its unique contribution from that of Musical Surprise. Indeed, the effects attributed to the high-level rhythmic tracking (St and Et model) cannot be explainable by low-level timing alone, as the St and Et regressors accounts for additional EEG variance beyond that explained by the preceding IOI. We now clarify this more explicitly in the manuscript (p. 13).

Moreover, to further address the reviewer’s comment, we also conducted a new control analysis for which we predicted the *subsequent* IOI instead of the preceding IOI (as reported above). A linear mixed model with the same factors revealed a trend in the effect of surprise level (χ²(1) = 3.52, p = 0.06), no main effect of condition (χ²(1) = 0.008, p = 0.90) and no interaction (χ²(1) = 0.70, p = 0.40), indicating that the bias is similar across real and shuffled music (Figure S3A, right panel). Thus, including the subsequent IOI as a regressor in the mTRF is possibly unnecessary. However, to further control for any potential issue, we re-run the main analysis adding the subsequent IOI as regressor in the mTRF. We thus run a full model with the following regressors: onset, spectral flux, inter-pitch interval, preceding IOI, subsequent IOI, Sp, Ep, St, Et, to which we subtracted the reduced model where St and Et were randomised (Figure S3B). The results confirm a unique contribution of St and Et features to the neural response in the real but not shuffled music beyond the contribution of subsequent and preceding IOI (the unique contribution of subsequent and preceding IOI is also shown in the topographies).

We have mentioned these considerations in the results (p. 6) and reported the stimuli analyses in the supplementary material (Figure S3, also pasted below).

**S3 Fig. A) Length of preceding and subsequent IOIs as a function of rhythmic surprise and condition.** Notes carrying high surprise are often preceded by relatively larger IOIs. A linear mixed model predicting the preceding IOI, with factors condition (real / shuffled) and surprise level (high / low), yielded no main effect of condition (χ2(1) = 1.12, p = 0.29), but a significant main effect of surprise level (χ2(1) = 16.89, p < .001), and an interaction of condition and surprise level (χ2(1) = 18.12, p < .001). This indicates that larger IOIs generally anticipate notes carrying high surprise, more so in real than in shuffled music (left panel). Conversely, the same analysis predicting the subsequent (rather than preceding) IOI, yielded a nearly-significant effect of surprise level (χ²(1) = 3.52, p = 0.06) but no main effect of condition (χ²(1) = 0.008, p = 0.90) and no interaction (χ²(1) = 0.70, p = 0.40). This indicates that notes carrying high surprise tend to be followed by larger IOIs, but comparably across real and shuffled music (right panel). **B) Unique contribution of St&Et is independent of preceding or subsequent IOI.**  To distinguish neural tracking of rhythm from spurious modulations of event-related potentials (ERPs) attributable to overlapping (i.e. temporally proximal) neural responses, we re-run the main analysis adding the length of the subsequent IOI as regressor in the mTRF. We thus run a full model with the following regressors: onset, spectral flux, inter-pitch interval, preceding IOI, subsequent IOI, Sp, Ep, St, Et. We then computed three reduced models, each randomising one of the following regressors: 1) St and Et, 2) preceding IOI, 3) subsequent IOI. The results of this control analysis confirm a unique contribution of St & Et features to the neural response in real but not shuffled music beyond the contribution of subsequent and preceding IOI. The plot shows the topographical maps representing group-average Δr resulting from the difference between full and the three reduced models across real and shuffled conditions.
