# Supplementary material for: Human newborns form musical predictions based on rhythmic but not melodic structure
Source: PLoS Biol. 2026 Feb 5;24(2):e3003600. doi: 10.1371/journal.pbio.3003600 (PMC12875487; doi:10.1371/journal.pbio.3003600)
Supplement: Attachment — Submitted filename: ResponseToReviewers_auresp_3.docx [file pbio.3003600.s019.docx]

We are glad that we have addressed the concerns of the reviewers and thank the editors for the opportunity to respond to the remaining suggestion from Reviewer 3. Below, we provide our responses in blue font to the reviewers’ comments (original comments in black font).

**Reviewer #1 (Benjamin Morillon):**

We congratulate the authors on this high-quality work.

We thank Reviewer 1 for their positive evaluation.

**Reviewer #2:**

I thank the authors for their careful and clear revisions and responses to reviewer comments. I feel the manuscript’s clarity and impact are improved and I have no further comments.

We thank Reviewer 2 for their constructive feedback.

**Reviewer #3 (Anne Kösem):**

The authors have satisfactorily addressed my concern. I suggest including the response to the reviewers that addresses how larger IOIs are associated with relatively more surprising notes (in music) in the main text (for example, in the results section, page 6). I believe this is an important point that is only currently indicated in the legend of Figure S3.

We have now incorporated this point into the main text in the Results section (page 6).
